# Supplementary material for: Pyphe, a python toolbox for assessing microbial growth and cell viability in high-throughput colony screens
Source: eLife. 2020 Jun 16;9:e55160. doi: 10.7554/eLife.55160 (PMC7297533; doi:10.7554/eLife.55160)
Supplement: Transparent reporting form [file elife-55160-transrepform.docx]

***eLife’s* transparent reporting form**

We encourage authors to provide detailed information *within their submission* to facilitate the interpretation and replication of experiments. Authors can upload supporting documentation to indicate the use of appropriate reporting guidelines for health-related research (see [EQUATOR Network](http://www.equator-network.org/%20)), life science research (see the [BioSharing Information Resource](https://biosharing.org/)), or the [ARRIVE guidelines](http://www.plosbiology.org/article/info:doi/10.1371/journal.pbio.1000412) for reporting work involving animal research. Where applicable, authors should refer to any relevant reporting standards documents in this form.

If you have any questions, please consult our Journal Policies and/or contact us: [editorial@elifesciences.org](mailto:editorial@elifesciences.org).

**Sample-size estimation**

- You should state whether an appropriate sample size was computed when the study was being designed
- You should state the statistical method of sample size computation and any required assumptions
- If no explicit power analysis was used, you should describe how you decided what sample (replicate) size (number) to use

Please outline where this information can be found within the submission (e.g., sections or figure legends), or explain why this information doesn’t apply to your submission:

As our study was not hypothesis-driven, no sample size estimation was used. One strength of the presented method is the ease with which thousands of colonies can be assayed in parallel, so we generally included many more replicates than would be standard practice (e.g. the data presented in Fig. 2 is based on ~20 replicates per strain).

**Replicates**

- You should report how often each experiment was performed
- You should include a definition of biological versus technical replication
- The data obtained should be provided and sufficient information should be provided to indicate the number of independent biological and/or technical replicates
- If you encountered any outliers, you should describe how these were handled
- Criteria for exclusion/inclusion of data should be clearly stated
- High-throughput sequence data should be uploaded before submission, with a private link for reviewers provided (these are available from both GEO and ArrayExpress)

Please outline where this information can be found within the submission (e.g., sections or figure legends), or explain why this information doesn’t apply to your submission:

Each figure legend and corresponding main text section states the number of replicates. The Methods section clearly describes how replicates were obtained for both data sets and that these were biological replicates in the sense that they came from different cryo-stocks.

Outliers were excluded from visualization (but not statistical analysis) in Fig. 3A and this is stated in the legend.

Quality filtering was done on the knock-out data set and the criteria are stated at the end of the corresponding Methods section.

**Statistical reporting**

- Statistical analysis methods should be described and justified
- Raw data should be presented in figures whenever informative to do so (typically when N per group is less than 10)
- For each experiment, you should identify the statistical tests used, exact values of N, definitions of center, methods of multiple test correction, and dispersion and precision measures (e.g., mean, median, SD, SEM, confidence intervals; and, for the major substantive results, a measure of effect size (e.g., Pearson's r, Cohen's d)
- Report exact p-values wherever possible alongside the summary statistics and 95% confidence intervals. These should be reported for all key questions and not only when the p-value is less than 0.05.

Please outline where this information can be found within the submission (e.g., sections or figure legends), or explain why this information doesn’t apply to your submission:

*Pyphe-interpret* was used to statistically assess differential fitness in media with and without phloxine B and its algorithms are described in detail in Appendix 3. Coefficients of variation (CVs) and fractions of unexplained variance (FUV) are reported in several places and are defined in the paragraph beginning at the bottom of p. 8. Other standard statistical methods (Pearson correlation, accuracy computation and linear regression) are used and referred to as such.

(For large datasets, or papers with a very large number of statistical tests, you may upload a single table file with tests, Ns, etc., with reference to sections in the manuscript.)

**Group allocation**

- Indicate how samples were allocated into experimental groups (in the case of clinical studies, please specify allocation to treatment method); if randomization was used, please also state if restricted randomization was applied
- Indicate if masking was used during group allocation, data collection and/or data analysis

Please outline where this information can be found within the submission (e.g., sections or figure legends), or explain why this information doesn’t apply to your submission:

Our study does not contain experimental groups and therefore no group allocation was done.

**Additional data files (“source data”)**

- We encourage you to upload relevant additional data files, such as numerical data that are represented as a graph in a figure, or as a summary table
- Where provided, these should be in the most useful format, and they can be uploaded as “Source data” files linked to a main figure or table
- Include model definition files including the full list of parameters used
- Include code used for data analysis (e.g., R, MatLab)
- Avoid stating that data files are “available upon request”

Please indicate the figures or tables for which source data files have been provided:

We have included some of the data underlying Figures 2-4 in the supplement. Providing raw data would not be useful nor feasible due to the nature of the data (images of plates for Figs 2 and 3, ImageStream images for Fig. 4). Data analysis is based on the pyphe toolbox which is publicly available at <https://github.com/Bahler-Lab/pyphe>. Parameters used for analysis are described in Methods.
